# CRHR1 antagonist alleviates LPS-induced depression-like behaviour in mice

**DOI:** 10.1186/s12888-023-04519-z

**Published:** 2023-01-09

**Authors:** Jie Sun, Lili Qiu, Hui Zhang, Zhiqiang Zhou, Lingsha Ju, Jiaojiao Yang

**Affiliations:** 1 Department of Anesthesiology, Zhongda Hospital, School of Medicine, Southeast University, Nanjing, Jiangsu China; 2 Department of Anesthesiology, Jinling Hospital, Medical College of Nanjing Medical University, Nanjing, China; 3 Department of Anesthesiology, Pain and Perioperative Medicine, The First Affiliated Hospital of Zhengzhou University, Zhengzhou, Henan China

**Keywords:** Depression-like behaviour, CRHR1, nectin3, Stress, HPA

## Abstract

**Background:**

Maladaptation of the HPA (hypothalamic–pituitary–adrenal) axis plays an important role in depression-like behaviour, but the specific molecular mechanisms are unknown. Here, we determined the roles of CRHR1 (corticotrophin releasing hormone receptor 1) and nectin3 in LPS (lipopolysaccharide)-induced depression-like behaviour in mice.

**Methods:**

C57BL/6 male mice were intraperitoneally injected with LPS (0.83 g/kg), and the open field, novelty-suppressed feeding, forced swimming, and tail suspension tests were performed after intraperitoneal injections of saline or antalarmin (20 mg/kg). The hippocampal mRNA levels of CRHR1 and nectin3 were determined by quantitative reverse transcription-PCR. The hippocampal protein levels of CRHR1, nectin3, and calbindin were measured by western blotting. The CORT (corticosterone) levels in the blood were measured by ELISA kits.

**Results:**

Antalarmin alleviated LPS-induced depression-like behaviour in male mice. Furthermore, antalarmin significantly inhibited changes in CRHR1, nectin3 and calbindin levels in the hippocampus and reduced the increase in CORT levels in LPS-treated mice.

**Conclusion:**

CRHR1antagonist showed antidepressant effects in LPS-induced depressive mice, and CRHR1/nectin3 signalling may play a crucial role in this process.

**Supplementary Information:**

The online version contains supplementary material available at 10.1186/s12888-023-04519-z.

## Introduction

Stress and its neurobiological correlates activate the HPA (hypothalamic–pituitary–adrenal) axis, resulting in the secretion of CRH (corticotrophin releasing hormone) from the hypothalamus and corticotrophin from the pituitary gland, followed by an increased synthesis and release of CORT (corticosterone) from the adrenal cortex. CRH is thought to be related to the development and progression of depression, accounting for many depression-related symptoms, such as insomnia, anxiety, psychomotor agitation, and decreased appetite [1]. CRH binds to CRHR1 (CRH receptor 1) in the hypothalamus, which is responsible for depression-like behaviour; therefore, inhibiting CRHR1 activity may provide novel psychopharmacological therapeutics for depression [2]. CAMs (synaptic cell adhesion molecules) include multiple families of proteins, such as nectins, neuroligins, and N-cadherin [3–5]. Previous studies have shown that CAMs are located at synaptic junctions, form interneuronal connections and modulate synapse formation, maturation or transmission, dynamically shaping synaptic plasticity [3, 6, 7]. Several CAMs are affected by stress, including nectin3, nectin1, and calbindin. Nectin3 is an immunoglobulin-like CAM that is primarily involved in synaptic plasticity in the hippocampus and stress-related cognitive impairment [8, 9]. Nectin3 has been found to be downregulated in the hippocampus following stress, and was accompanied by activation of the HPA axis [9].

Lipopolysaccharides (LPSs) are primarily composed of gram-negative bacteria (e.g., *Escherichia coli* (*E. coli*)), and their administration to animals is a common model for depression. LPS elicits an immune response in mice, which causes symptoms such as weight loss, decreased eating and drinking and decreased locomotor activity [10]. At the same time, LPS administration increases CORT levels, indicating that LPS injection might induce activity in the HPA axis [11]. In this study, we established a model of depression induced by 0.83 g/kg LPS, which has been validated by several studies [11, 12]. Although some studies have shown that CRHR1/nectin3 plays an important role in stress-induced cognitive deficits, we still do not know whether it has effects on LPS-induced depression-like behaviour [8, 9, 13]. Therefore, we investigated the effects of CRHR1/nectin3 on LPS-induced depression-like behaviour and evaluated whether the CRHR1 antagonist antalarmin alleviated the depression-like behaviour and molecular changes induced by LPS administration.

## Materials and methods

### Animals

Male C57BL/6 N mice (*n* = 58, 7-8 weeks old; Animal Center of Southeast University, Nanjing, China) were maintained at a constant temperature (22 ± 1 °C) under a 12-h light/dark cycle (lights on at 8:00 am). The mice had free access to food and chow, and there were 4-5 mice in each cage. All animal experiments were conducted in accordance with the National Institutes of Health Guide for the Care and Use of Laboratory Animals.

### Model of depression and experimental groups

**Fig. 1:**
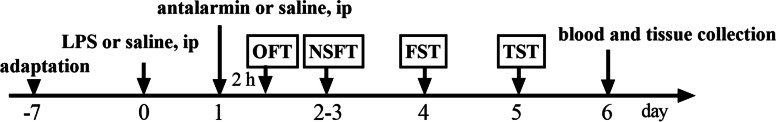
Timeline of drug injection, behavioural tests, and tissue collection. See text for details. OFT, open field test; NSFT, novelty-suppressed feeding test; FST, forced swimming test; TST, tail suspension test

To establish a model of depression, the mice were intraperitoneally injected with LPS (0.83 g/kg; Sigma Aldrich, Shanghai, China), as previously described [11, 12]. The mice were habituated for 7 days after arrival and then randomly divided into control (CON), antalarmin (ANT, a CRHR1 antagonist; 20 mg/kg; Tocris Bioscience, Bristol, UK), LPS and antalarmin plus LPS (LPS-ANT) groups. On the first day (D0), the mice in the LPS and LPS-ANT groups were intraperitoneally injected with LPS, while the mice in the CON and ANT groups were injected with saline. Twenty-four hours after LPS injection (D1), the mice in the ANT and LPS-ANT groups were intraperitoneally injected with antalarmin, and the mice in the CON and LPS groups were injected with saline. These doses of LPS and antalarmin were chosen based on previous studies [11, 12, 14]. No toxic effects have been observed at this dose of antalarmin [14–16]. The behavioural tests were investigated from D1 to D5, as shown in Fig. 1. At the day after behavioural test, mice were anesthetized by intraperitoneal injection of 2% sodium pentobarbital (60 mg/kg; Sigma Aldrich, St Louise, MO, USA), and the brains were removed for PCR (*n* = 4-5/group) and western blot (*n* = 3/group) analysis.

### Behavioural tests

All of the behavioural tests were performed between 09:00 and 16:00. The behaviour during the open field test was recorded and analysed using software purchased from Shanghai Softmaze Information Technology Co., Ltd. (XR-XZ301, China). A video camera was used to record the novelty-suppressed feeding, forced swim, and tail suspension tests, and a well-trained investigator who was blinded to the aim of the study performed the behavioural experiments. The open field, novelty-suppressed feeding, forced swim, and tail suspension tests were used to evaluate depression-like behaviour. Although the open field and novelty-suppressed feeding tests are usually regarded as anxiety-like behavioural tests, they were regarded as depression-like behavioural tests in some studies [17, 18]. LPS-induced depression-like behaviour can be observed 24 h after LPS administration, so we conducted behavioural tests 26 h after LPS injection [12, 19].

#### Open field test (OFT)

The OFT was carried out in a chamber (50 cm × 50 cm × 50 cm; length × width × height) made of white polyvinyl chloride. A mouse was placed in the centre of the arena and was free to explore for 5 minutes. A video camera that tracked the movement of each mouse was placed directly above the chamber. The arena was cleaned with 75% ethanol after each test to prevent residual olfactory cues. The total travelled distance and the time spent in the central area were measured to evaluate the anxiety- and depression-like behaviour of mice [17, 18].

#### Novelty-suppressed feeding test (NSFT)

A plastic box (50 cm × 50 cm × 50 cm; length × width × height) was used to perform the NSFT, and the floor of the box was covered with approximately 2 cm of saw dust bedding. All food was removed from the home cage 24 hours before the NSFT. During testing, the investigator placed a single pellet of food in the centre of the box and then placed the mouse in a corner of the box. A stopwatch was started when the mouse was placed, and the latency to eat was timed. The latency was defined as the amount of time between the mouse being placed and the mouse biting the pellet. Immediately afterwards, the animal was placed back in its home cage, and the amount of food consumed within 5 min was divided by its weight (home cage food consumption index). The arena was cleaned with 75% ethanol after each test to prevent residual olfactory traces.

#### Forced swim test (FST)

The mice were individually placed in a cylinder (20 cm × 30 cm; diameter × height) of water (temperature of 23–25 °C; depth of 15 cm) and swam for 6 min. When the mouse finished the test, it was transferred to its original cage after its body was wiped with a dry towel. The animal behaviours were recorded from the side of the cylinder. The immobility score was assessed during the final 4 minutes of the test session. Immobility was defined as the time that the mouse passively floated with no additional activity except movement that maintained its balance in the water. The water was replaced at the end of each test.

#### Tail suspension test (TST)

The subjects were recorded for 6 minutes. The animals were suspended by their tail, and tape was used to secure them to a horizontal bar. The behavioural apparatus was thoroughly cleaned with 75% ethanol after each test. The immobility time was defined as the amount of time that the mouse hung passively and was completely motionless except for breathing.

### Western blot measurements

The entire hippocampus was rapidly dissected from the brain on ice and stored at − 80 °C after the last behavioural test. The hippocampal tissues were homogenized in RIPA (radioimmunoprecipitation assay) lysis buffer mixed with a 1% protease inhibitor cocktail and 1% phenylmethanesulfonyl fluoride. The supernatant was collected after centrifugation at 13,000 x g for 10 min at 4 °C, and the protein concentration was measured by BCA protein assay kit (Beyotime, China). Samples (forty micrograms of protein per lane) were loaded on SDS–PAGE gels and subsequently transferred to polyvinylidine fluoride membranes. Some waste samples were placed on the edge of the membrane to rule out edge effects. The membranes were incubated overnight with primary antibodies at 4 °C after blocking with 3% bovine serum albumin in TBST (Tris-buffered saline with Tween) for 1 h at room temperature. The following antibodies were used: rabbit anti-CRHR1 (1:1000, ab229585, Abcam, Cambridge, UK), rabbit anti-nectin3 (1:5000, ab63931, Abcam, Cambridge, UK), rabbit anti-calbindin (1:20,000, CB-38, Swant, Marly, Switzerland) and mouse anti-β-actin (1:1000; ab8226, Abcam, Cambridge, UK). After washing in TBST three times, the membranes were incubated at room temperature (1 h) with horseradish peroxidase-conjugated secondary antibodies (1:7000, Bioworld Technology, St. Louis Park, MN, USA). The chemiluminescence method was used to detect the protein bands. The bands were quantified by ImageJ software (National Institutes of Health, Bethesda, MD, USA). All results were normalized by taking the value of the CON group as 100%.

### Quantitative mRNA measurements

All hippocampal tissues were individually placed in tubes containing RNAlater solution (Invitrogen, Carlsbad, CA) and stored at − 20 °C. RNA was extracted from the samples using a Purelink® RNA Mini Kit (Invitrogen, Valencia, CA), reverse-transcribed with a high-capacity cDNA reverse transcription kit (Hiscript® III RT Supermix for qPCR; Vazyme, China), and then analysed by qRT–PCR (reverse transcription-PCR) in a QuantStudio 6 Flex Real-Time PCR System (Applied Biosystems, Foster City, CA) with a master mix kit (ChamQ Universal SYBR qPCR master mix; Vazyme, China). We analysed the mRNA expression levels of CRHR1 and nectin3 in the hippocampus, with GAPDH serving as an internal control. The primer sequences for CRHR1, nectin3 and GAPDH are listed in Table 1. The ΔΔCT method was used to calculate the gene expression, and the data are presented as the relative fold change from the CON group.

**Table 1:** Primer sequences of CRHR1, nectin3 and GAPDH

Gene	Forwards	Reverse
CRHR1	GGGCAGCCCGTGTGAATTATT	ATGACGGCAATGTGGTAGTGC
Nectin3	GAAGGCGAATTACTTGTGTTGTAA	TCCATCATATCCTGTTACTAAACTT
GAPDH	CAGTGGCAAAGTGGAGATTGTTG	TCGCTCCTGGAAGATGGTGAT

### Measurement of CORT serum levels

We collected trunk blood from 9:00-11:00 am on the day after the last behavioural test to assess the changes in serum corticosterone levels. In detail, the mice were anaesthetized with 2% sodium pentobarbital in saline (50 g/kg, i.p.; Sigma, St Louise, MO, USA), and trunk blood was collected immediately after decapitation. The anaesthesia, decapitation and blood collection were completed in 5 minutes. The samples were centrifuged at 1000 x g for 15 min, and the supernatants were collected and frozen at − 80 °C until assayed for CORT. The CORT concentrations were quantified using ELISA kits (Cayman Chemical Company, Ann Arbor, MI, USA) following the manufacturer’s instructions.

### Statistical analyses

Data are presented as the mean ± SEM (standard error of the mean). GraphPad Prism 8.0 software (GraphPad, San Diego, CA, USA) was used to analyse the raw data. We used the Shapiro–Wilk test to determine the normality of all the data. If the data were normally distributed, an ANOVA was applied; otherwise, a nonparametric test was used. One-way ANOVA was used for group comparisons, followed by Bonferroni post hoc tests when appropriate. For the weight and behaviour analyses, two-way ANOVA followed by Bonferroni’s multiple comparisons test was used. Differences among groups with *P* < 0.05 were considered statistically significant.

## Results

### Body weight

**Fig. 2:**
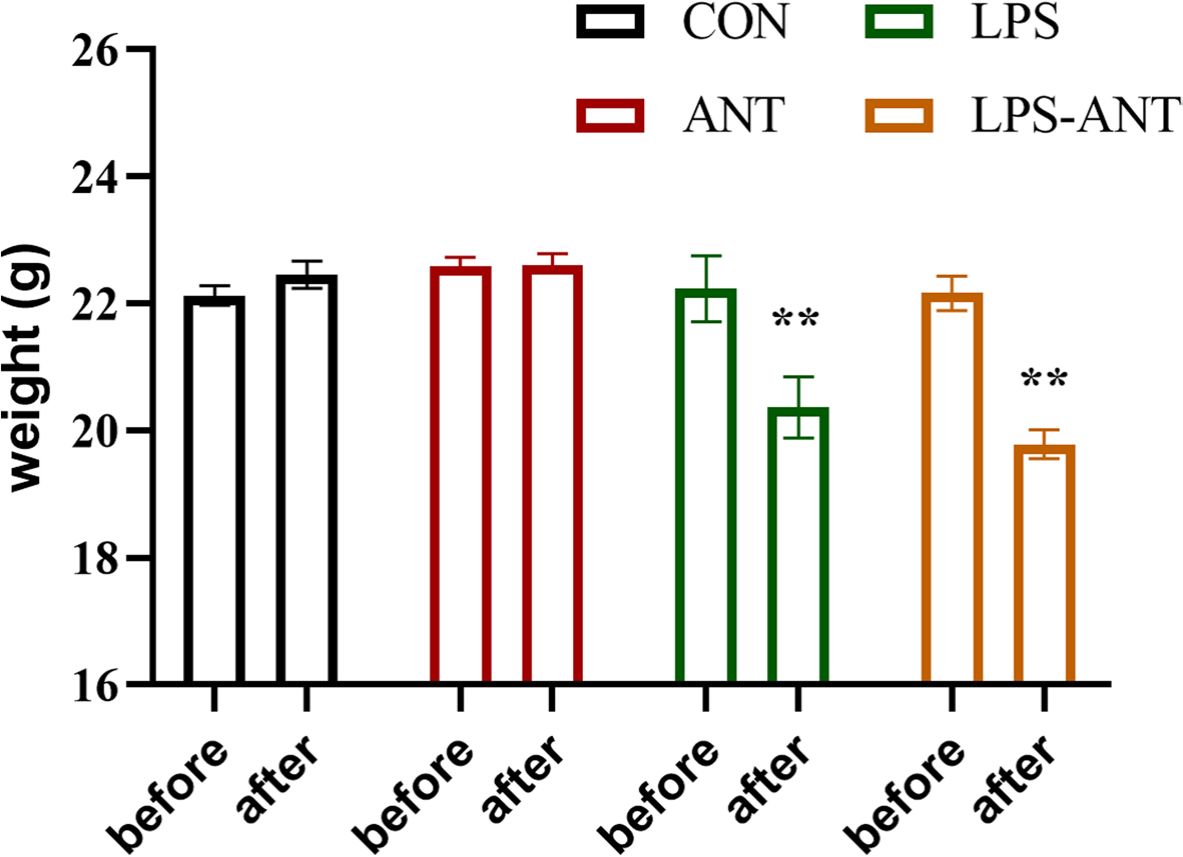
The effects of LPS on body weight. Histograms show the body weights in the four groups before and after LPS injection. Data are presented as the mean ± SEM (*n* = 15 mice in the CON and LPS groups; *n* = 14 mice in the ANT and LPS-ANT groups). *P < 0.05 and **P < 0.01 vs. the CON group

In the present study, body weight was taken immediately before the behavioural testing started. There were statistically significances on time factor, treatment factor and interaction between time and treatment on body weight (time factor: F_(1, 108)_ = 19.45, *P* < 0.01; treatment factor F_(3, 108)_ = 12.17, P < 0.01; interaction: F_(3, 108)_ = 9.31, *P* < 0.01; Fig. 2). Bonferroni’s multiple comparisons test showed that the body weights in the LPS group and LPS-ANT group were significantly lower than those in the CON group after LPS injection (*P* < 0.05). There were no differences between groups in terms of body weight before LPS injection (*P* > 0.05).

### Antalarmin alleviated the depression-like behaviour induced by LPS administration

**Fig. 3:**
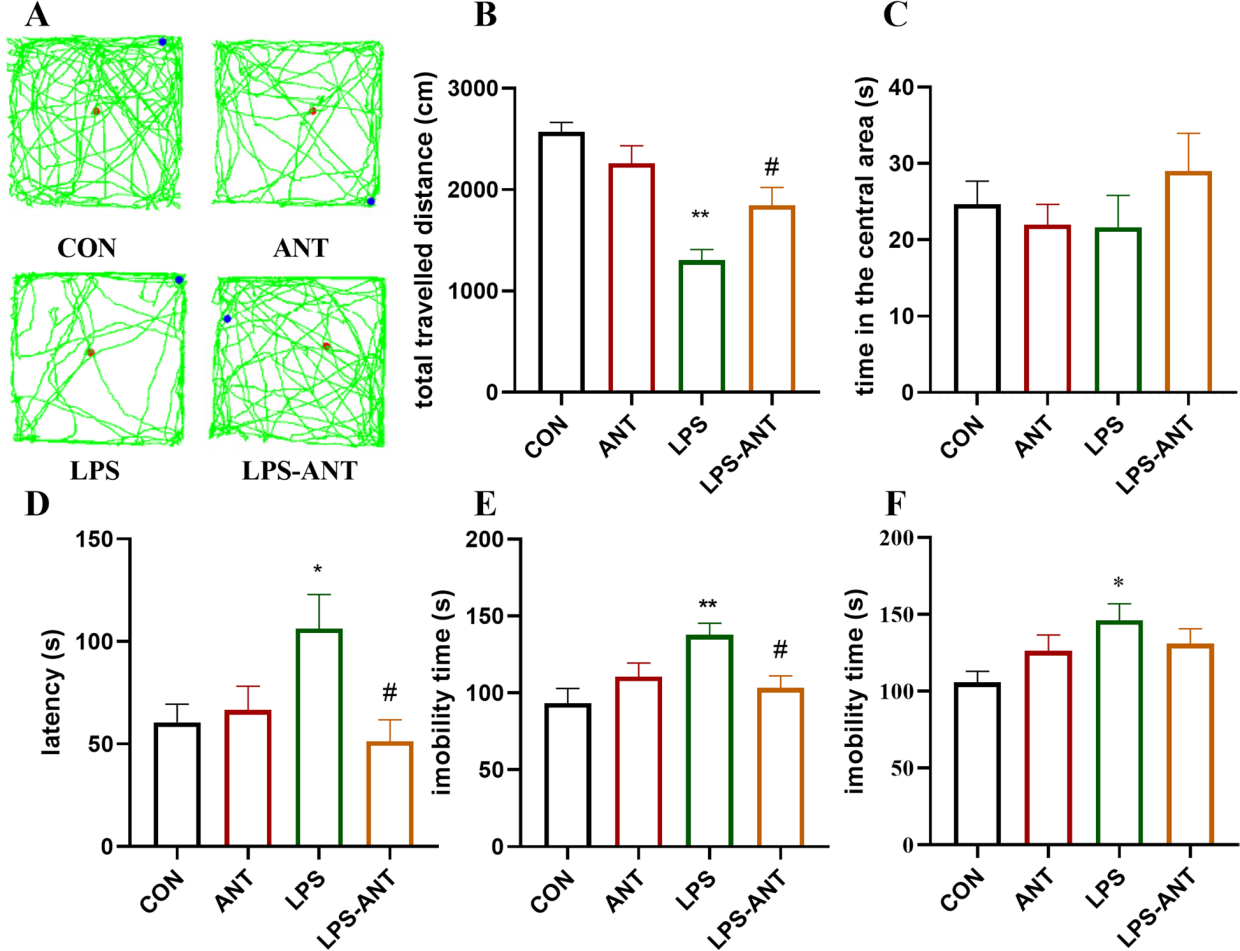
Effects of antalarmin on depression-like behaviour induced by LPS administration. **A** Representative graphs of trajectories during the open field test (OFT). **B**-**C** Histograms showing the total travelled distance and the time spent in the central area in the open field test (OFT). **D** Histogram showing the feeding latencies after fasting in the novelty-suppressed feeding test (NSFT). **E**-**F** Histogram showing the immobility time of the mice in the forced swimming test (FST) and the tail suspension test (TST). Data are presented as the mean ± SEM (*n* = 15 mice in the CON and LPS groups; *n* = 14 mice in the ANT and LPS-ANT groups). *P < 0.05 and **P < 0.01 vs. CON group; #*P* < 0.05 and ##*P* < 0.01 vs. LPS group

In the OFT, there were statistically significances on LPS effect and but not antalarmin effect in the total travelled distance (LPS effect: F_(1, 54)_ = 36.84, *P* < 0.01; antalarmin effect F_(1, 54)_ = 0.70, *P* > 0.05; Fig. 3A, B). There was also a statistically significant interaction between LPS/control and antalarmin/vehicle in the total travelled distance (F_(1, 54)_ = 9.46, *P* < 0.01; Fig. 3A, B). Bonferroni’s multiple comparisons test found that the total travelled distance was significantly lower in the LPS group than in the CON group (P < 0.01), and antalarmin alleviated LPS-induced decreases in total travelled distance (*P* = 0.047). There were no statistically significances on LPS effect, antalarmin effect or interaction between LPS/control and antalarmin/vehicle on the time spent in the central area (LPS effect: F_(1, 54)_ = 0.28, *P* > 0.05; antalarmin effect F_(1, 54)_ = 0.38, *P* > 0.05; interaction: F_(1,54)_ = 1.72, *P* > 0.05; Fig. 3C). In the NSFT, there were no statistically significances on LPS effect and antalarmin effect (LPS effect: F_(1, 54)_ = 1.51, *P* > 0.05; antalarmin effect F_(1, 54)_ = 3.90, *P* > 0.05; Fig. 3D). There was a statistically significant interaction between LPS/control and antalarmin/vehicle for the latency to eat (F_(1,54)_ = 6.06, *P* = 0.02; Fig. 3D), but it did not alter the home cage food consumption index (F_(1,54)_ = 0.28, *P* > 0.05, data not shown). Bonferroni’s multiple comparisons test found that mice in the LPS group had a higher latency to eat than mice in the CON group (*P* = 0.049) and LPS-ANT group (*P* = 0.01). In the FST, there were statistically significances on LPS effect but not antalarmin effect for the immobility time (LPS effect: F_(1, 54)_ = 4.76, *P* = 0.03; antalarmin effect F_(1, 54)_ = 1.03, *P* > 0.05; Fig. 3E). There was a statistically significant interaction between LPS/control and antalarmin/vehicle for the immobility time (F_(1,54)_ = 9.33, *P* < 0.01; Fig. 3E). Bonferroni’s multiple comparisons test found that mice in the LPS group had higher immobility times than mice in the CON group (*P* < 0.01) and LPS-ANT group (*P* = 0.03). In the TST, there were no statistically significances on LPS effect but not antalarmin effect or interaction between LPS/control and antalarmin/vehicle for the immobility time (LPS effect: F_(1, 54)_ = 5.42, *P* = 0.02; antalarmin effect F_(1, 54)_ = 0.07, *P* > 0.05; interaction: F_(1,54)_ = 1.72, *P* > 0.05; interaction: F_(1,54)_ = 3.41, *P* > 0.05, Fig. 3F). Bonferroni’s multiple comparisons test revealed that the immobility time in the LPS group was significantly higher than that in the CON group (*P* = 0.02) but not higher than that in the LPS-ANT group (*P* > 0.05).

### Antalarmin reversed the hippocampal CRHR1/nectin3 mRNA changes induced by LPS administration

**Fig. 4:**
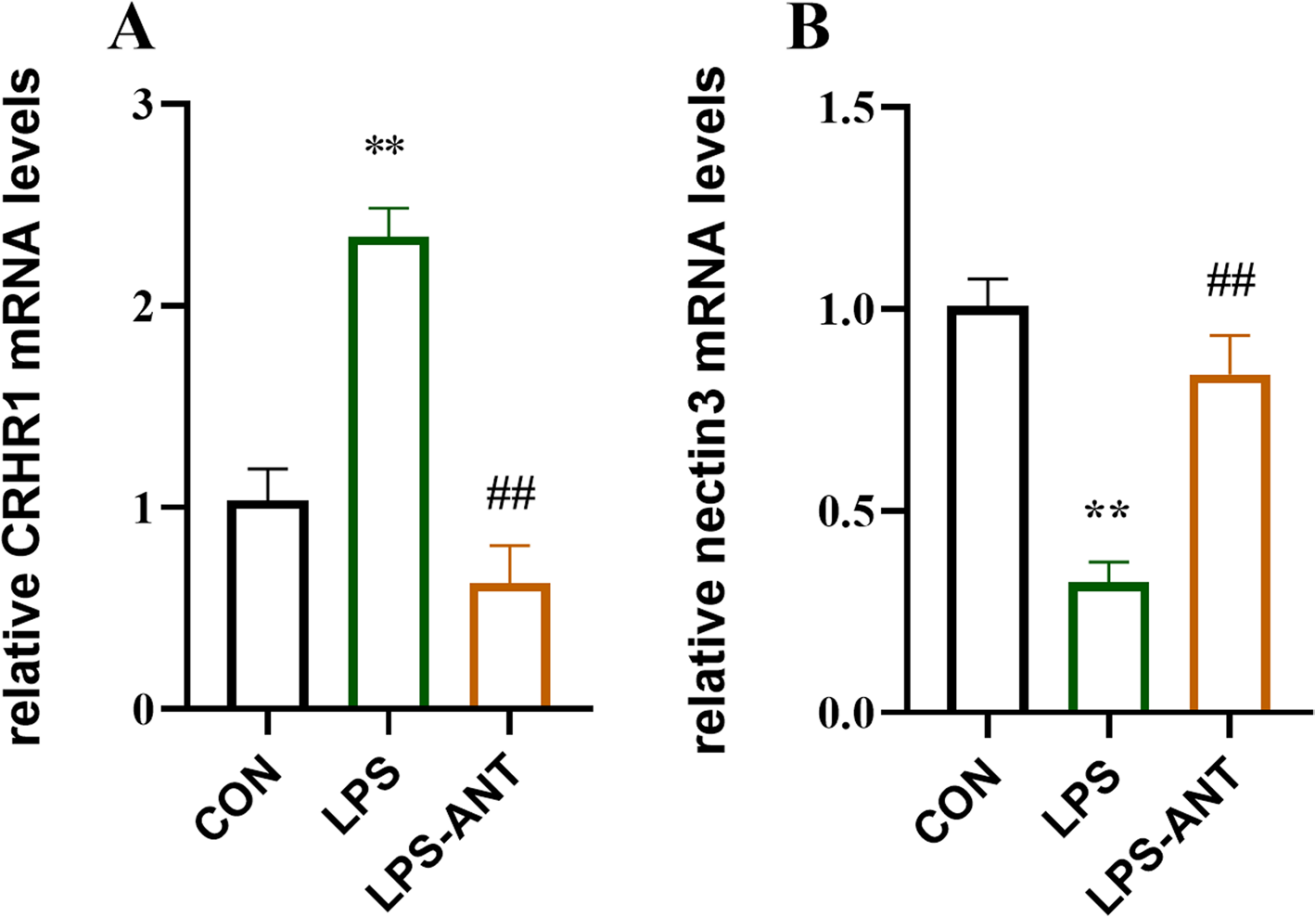
Hippocampal CRHR1 and nectin3 mRNA levels in response to the antidepressant-like activity of antalarmin. **A**, **B** Hippocampal mRNA levels of CRHR1 and nectin3 in the mice. Data are presented as the mean ± SEM, with 4 mice/group. *P < 0.05 and **P < 0.01 vs. CON group; #*P* < 0.05 and ##*P* < 0.01 vs. LPS group

In the hippocampal gene transcription measurement, there was a statistically significant between-subjects effect of treatment on the CRHR1 mRNA levels (F_(2,9)_ = 30.62, *P* < 0.01; Fig. 4A). Bonferroni’s multiple comparison analysis showed that the CRHR1 mRNA levels in the LPS group were increased compared with those in the CON group (*P* < 0.01) and LPS-ANT group (*P* < 0.01). Similarly, there was a statistically significant between-subjects effect of treatment on nectin3 mRNA levels in the hippocampus (F_(2,9)_ = 23.56, *P* < 0.01; Fig. 4B). Bonferroni’s multiple comparison analysis revealed that the nectin3 mRNA levels in the LPS group were decreased compared with those in the CON group (*P* < 0.01) and LPS-ANT group (*P* < 0.01).

### Antalarmin reversed the LPS-induced changes in the hippocampal protein levels of CRHR1, nectin3 and calbindin

**Fig. 5:**
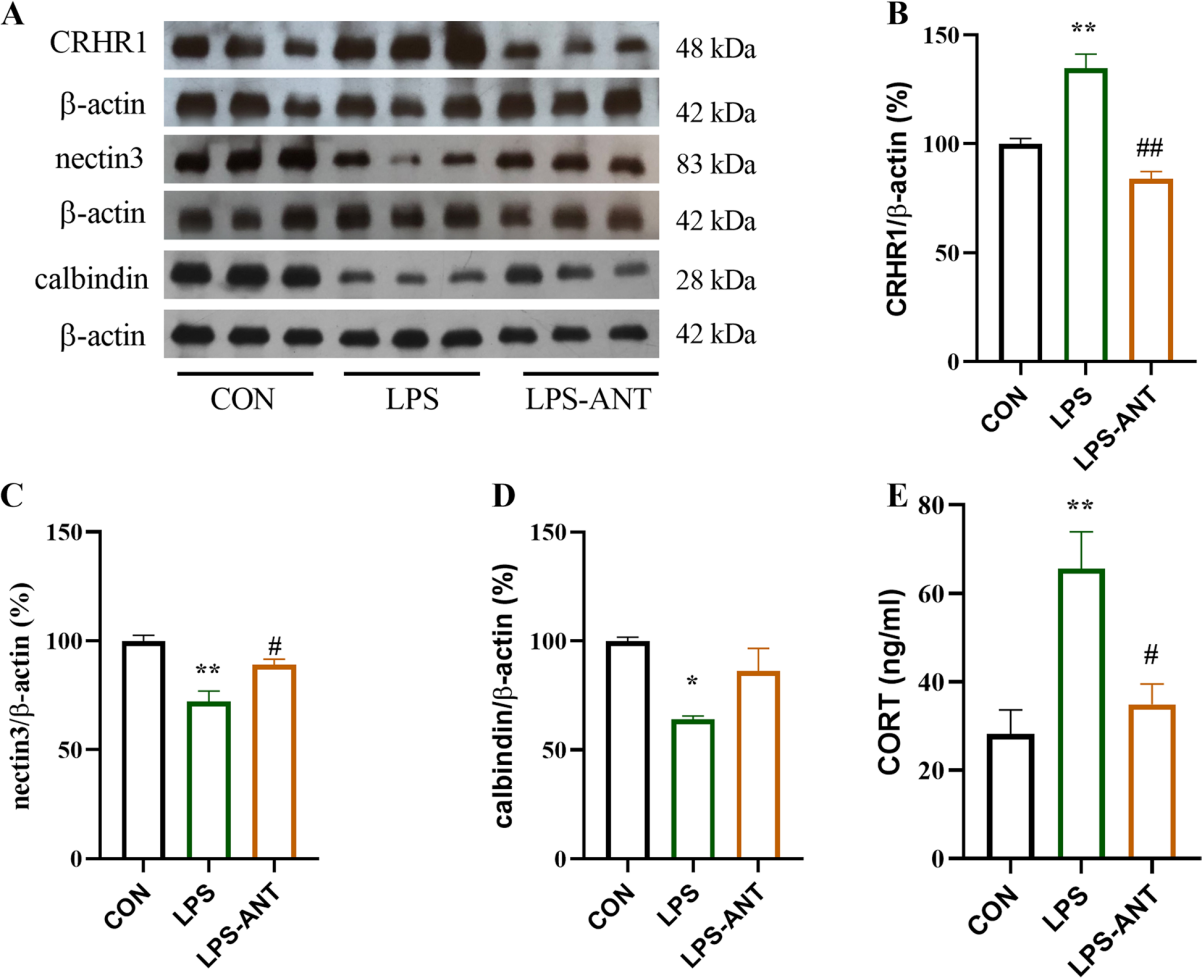
Role of hippocampal CRHR1, nectin3, calbindin protein levels and CORT levels in the antidepressant-like activity of antalarmin. **A** Representative blots of the protein levels in the three groups. **B**–**F** Protein levels of CRHR1, nectin3, and calbindin in the hippocampus of male mice. Data normalized with respect to the CON group are presented as the mean ± SEM, with 3 mice/group. CORT levels in the blood of male mice. Data are presented as the mean ± SEM, with 8 mice/group. *P < 0.05 and **P < 0.01 vs. CON group; #*P* < 0.05 and ##*P* < 0.01 vs. LPS group

We determined the hippocampal protein levels of CRHR1, nectin3 and calbindin, a downstream molecule. We found that there were statistically significant between-subjects effects of treatment on hippocampal CRHR1 protein levels (F_(2,6)_ = 34.22, *P* < 0.01; Fig. 5A, B). Bonferroni’s multiple comparison analysis showed that hippocampal CRHR1 protein levels in the LPS group were significantly higher than those in the CON group (*P* < 0.01). Antalarmin significantly reduced the levels of CRHR1 in the hippocampus (*P* < 0.01). There were also statistically significant between-subjects effects of treatment on nectin3 (F_(2,6)_ = 16.30, *P* < 0.01; Fig. 5A, C) and calbindin protein levels (F_(2,6)_ = 8.77, *P* = 0.02; Fig. 5A, D). Bonferroni’s multiple comparison analysis showed that the hippocampal nectin3 protein levels in the LPS group were significantly lower than those in the CON group (*P* < 0.01) and LPS-ANT group (*P* = 0.03). Furthermore, Bonferroni’s multiple comparison analysis revealed that the calbindin protein levels in the hippocampus in the LPS group were significantly lower than those in the CON group (*P* = 0.02) but not lower than those in the LPS-ANT group (*P* > 0.05). However, there were no differences in calbindin protein levels between the CON group and the LPS-ANT group (*P* > 0.05). These western blot results were consistent with the PCR results.

### Antalarmin reversed the increase in CORT levels induced by LPS administration

To determine whether the increased CRH and CRHR1 levels were related to the activity of the HPA axis, we measured CORT levels in the blood on D6. We found that there were statistically significant between-subjects effects of treatment on the levels of CORT (F_(2,21)_ = 9.93, *P* < 0.01, Fig. 5E). Bonferroni’s multiple comparison analysis showed that the CORT levels in the LPS group were significantly lower than those in the CON group (*P* < 0.01) and LPS-ANT group (*P* < 0.01).

## Discussion

In this work, we found that antalarmin was effective against LPS-induced depression-like behaviour. To establish models of depression, several methods have been used in animal research, including intraperitoneal injection of LPS and chronic restraint stress [12, 20]. Previous studies have demonstrated that intraperitoneal injection of LPS can induce behavioural changes within 1 week in mice [21, 22]. In this study, the mice that were administered LPS showed depression-like behaviour within 1 week, such as decreased total travelled distance in the OFT, increased latency to eat in the NSFT and increased immobility time in the FST and TST; these behaviours were accompanied by HPA axis activation and decreased levels of nectin3 and calbindin. Maladaptation of the HPA axis is thought to play an important role in stress-induced neuropsychiatric disorders; however, the potential molecular mechanisms have not yet been explained. Overexpression of CRH and CRHR1 can be regarded as the primary neurobiological correlate of major depressive disorder [23]. A recent study demonstrated that stress suppressed calbindin levels in the hippocampus through the CRHR1/nectin3 system, and calbindin levels were thought to be involved in stress-related spatial memory impairments [24]. In this study, we focused on the CRHR1/nectin3 system, as it may be a possible mechanism underlying depression-like behaviour.

We used an inflammation-induced depression-like behaviour model that has been validated in numerous studies [12, 25]. Weight loss is a common side effect of LPS administration, and there are currently few treatments available. However, weight loss within 2 weeks is clinically regarded as a measure of depression [26]. Here, we found that LPS administration resulted in similar weight loss when it was evaluated immediately before the behavioural testing started. In this study, the mice showed depression-like behaviour within 5 days after LPS administration, which was prevented by antalarmin administration. Considering the strength of the LPS effect, some studies behavioural tests were done only 1 day following LPS injection, but other studies found behavioural changes within 5 days, which were similar with our study [19, 21, 22]. It is reported depression-like behaviour 24 h after LPS injection, so we administered the CRHR1 antagonist antalarmin (20 mg/kg) 1 day after LPS injection, when LPS had induced the depression-like behaviour [19, 21]. Antalarmin is a nonpeptide CRHR1 receptor antagonist that can penetrate the blood–brain barrier and inhibit CRH binding in the hippocampus; antalarmin may alleviate LPS-induced increases in CRHR1 levels, and this effect could be observed 2 hours after antalarmin injection [15, 16, 27]. However, antalarmin may have short-term effects, because there were no differences in the TST test between the LPS and LPS-ANT groups 4 days after its injection, which explains why some studies need several injections of antalarmin [15]. Many of the changes, such as inflammation, kynurenine and activation of the HPA axis, could be observed within the first 24 hours after LPS injection [12, 16]. CRH and its receptors have fundamental neurobiological relationships with behavioural responses to stress in major depressive disorder [23]. Hippocampal CRH is mainly expressed in inhibitory interneurons, and it interacts primarily with CRHR1 in pyramidal neurons [13]. Acute stress usually causes an increase in CRH levels in the hippocampus, which affects CRHR1 levels and leads to reduced spine density and memory defects [28]. Additionally, chronic exposure could reduce the complexity of dendrites of CA3 neurons, which could be abolished by inhibiting hippocampal CRHR1 [13]. Recent evidence has also suggested that increased CRH–CRHR1 signalling in the hippocampus mediates the negative effects of early-life stress and leads to impairments in dendritic development and synaptogenesis, as well as cognitive deficits [29]. These findings suggest that sustained CRH-CRHR1 signalling activation during stress may promote dendritic remodelling. CRHR1 modulates the adverse effects of stress on synaptic plasticity and cognition and might be a key mediator of neuroendocrine and behavioural responses to stress [9]. Furthermore, the paraventricular nucleus of the hypothalamus (PVN) receives input from limbic systems (such as the hippocampus, amygdala and bed nucleus of the stria terminalis) as a result of psychological and physical stressors. Hippocampal CRHR1 has also been shown to promote the activation of the HPA axis [30]. Here, we found increased expression of hippocampal CRHR1 and serum corticosterone after 6 days of LPS injection, indicating activation of the HPA axis. However, antalarmin, a CRHR1 antagonist, blocked the activity of the HPA axis.

**Fig. 6:**
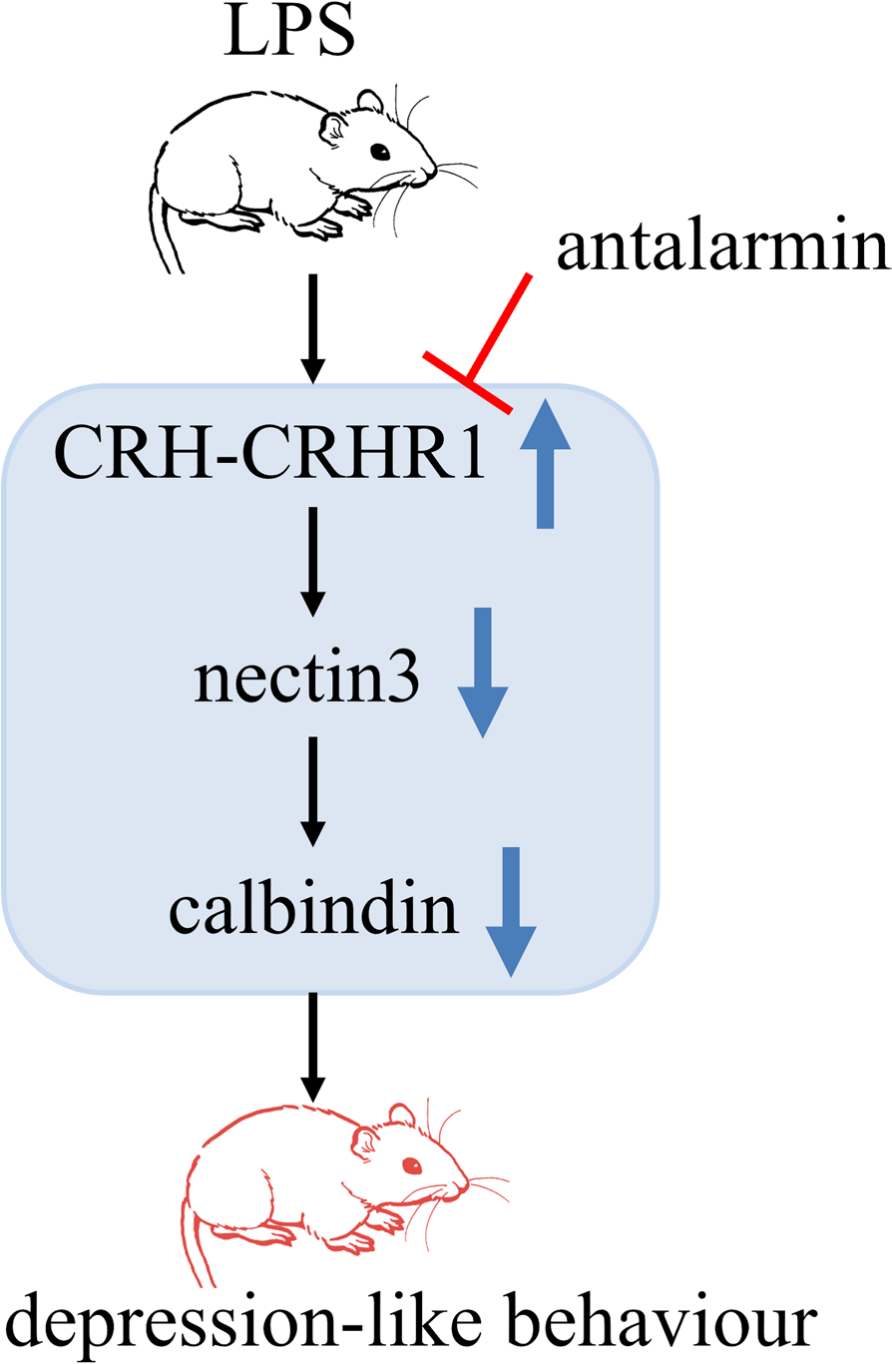
General overview of the mechanisms of this study. LPS induced depression-like behaviour in male mice by increasing the expression of CRHR1 and decreasing the expression of nectin3 and calbindin; however, antalarmin (CRHR1 antagonist) alleviated the above changes

Nectin3 is an immunoglobulin-like CAM that is expressed throughout the cell membrane. Nectin3 is primarily located at adherens junctions in the hippocampus and plays an important role in synaptic plasticity and stress-related cognitive disorders [24, 31]. Hippocampal nectin3 has been found to be downregulated in both acute stress and chronic stress, which indicates that it is involved in CRH-CRHR1 signalling and stress-induced cognitive alterations [8, 9]. Moreover, stress has been shown to suppress hippocampal calbindin levels via the CRHR1/nectin3 system, which showed increased CRHR1 and decreased nectin3 levels simultaneously [24]. Calbindin is a calcium-binding protein that is found in both dendritic spines and axonal boutons and modulates intracellular Ca2+ dynamics and synaptic plasticity [24]. Dysregulation of calbindin, specifically decreases in calbindin-expressing interneurons in the neocortex, has been linked to stress-related psychiatric disorders [32–34]. The cell adhesion molecule nectin3 is able to interact with calbindin in hippocampal neurons, which demonstrates that nectin3 is an upstream modulator of calbindin [24]. In this study, we collected the tissues after behavioural tests and we found that LPS induced the activation of the HPA axis, which led to increased blood corticosterone and increased expression of hippocampal CRHR1. The increased CRHR1 was accompanied by a simultaneous decrease in nectin3, which may lead to decreased calbindin expression in the hippocampus, and the CRHR1 antagonist could alleviate the above changes. This confirmed that CRHR1/nectin3 signalling may be involved in LPS-induced depression-like behaviours (Fig. 6).

There are some limitations in the study. One limitation is that we did not detect the effect of antalarmin on the biochemical changes. However, some laboratory evidence suggests that there were no obvious effects of antalarmin itself [14–16]. Moreover, the behavioural results of our study were the same as those of other studies, which showed no effects on depression-like behaviour in animals treated with antalarmin alone [15, 35]. The second limitation is that we used the FST to evaluate depression-like behaviour. The FST was doubted recently because it is difficult to know the real reason that the mice stop swimming. However, the FST is still a crucial test for depression-like behaviour, and there are no more accurate, effective or practical methods to replace it [36]. The third limitation is that some studies have shown that the dorsal hippocampus is more related to depression-like behaviour, while we only detected whole hippocampal gene/protein expression [37]. Although the whole hippocampus may largely indicate the effects of the dorsal hippocampus, we still need to focus on the dorsal hippocampus in future research.

In summary, the results demonstrated that a CRHR1 antagonist alleviates LPS-induced depression-like behaviour in male mice by inhibiting CRHR1/nectin3 signalling, which further increased the expression of calbindin. Although more studies are needed to elucidate the detailed mechanisms, the current results provide critical insights into CRHR1/nectin3 signalling in depression-like behaviour.

## Supplementary Information

**Additional file 1.**

**Additional file 2.**

**Additional file 3.**

## Data Availability

The datasets analyzed during the current study are not publicly available but are available from the corresponding author on reasonable request.
