# Supplementary figures and images for: CRHR1 antagonist alleviates LPS-induced depression-like behaviour in mice
Source: BMC Psychiatry. 2023 Jan 9;23:17. doi: 10.1186/s12888-023-04519-z (PMC9830857; doi:10.1186/s12888-023-04519-z)

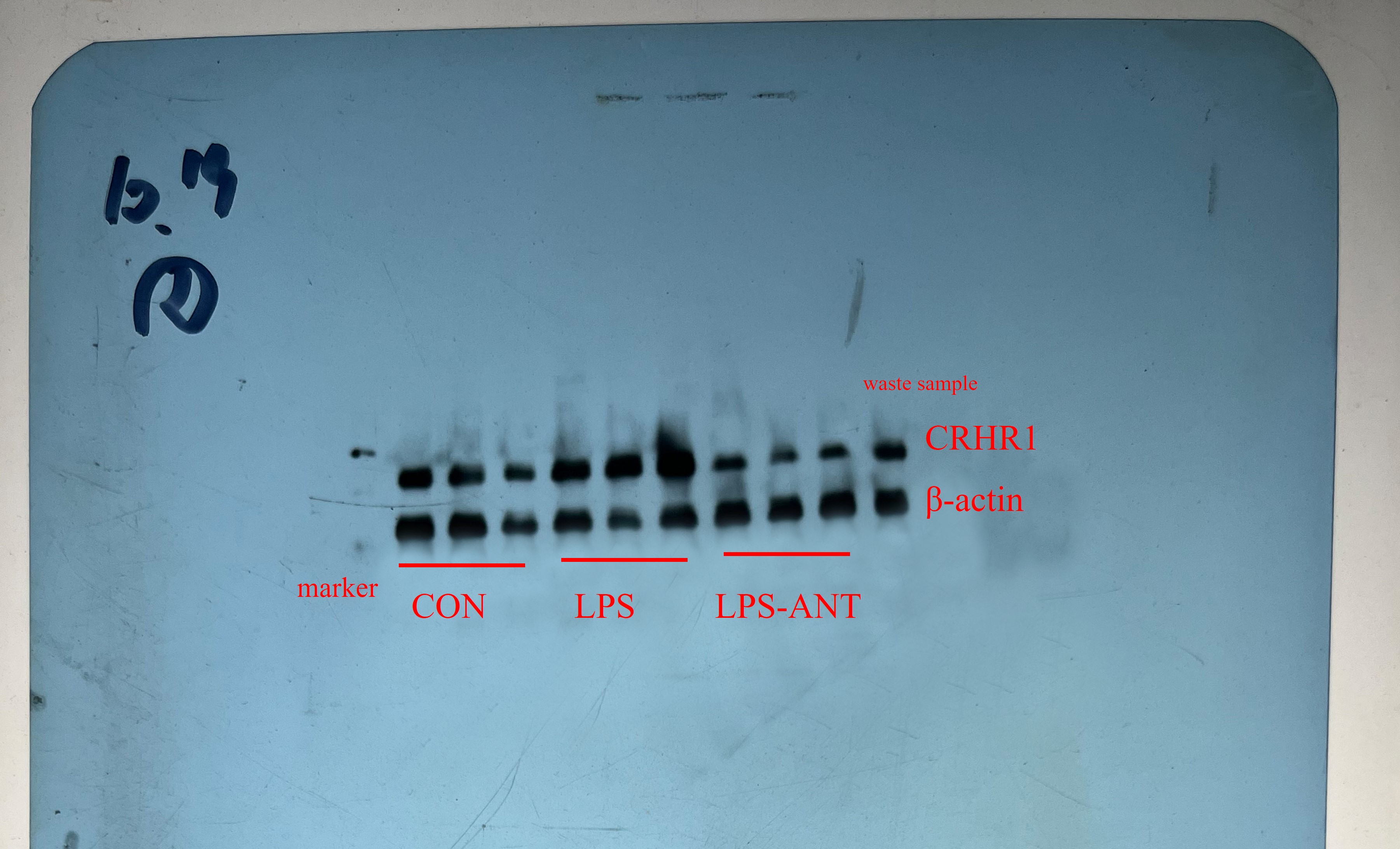

Supplement: Supplementary file 1 — Additional file 1. [file 12888_2023_4519_MOESM1_ESM.jpg]

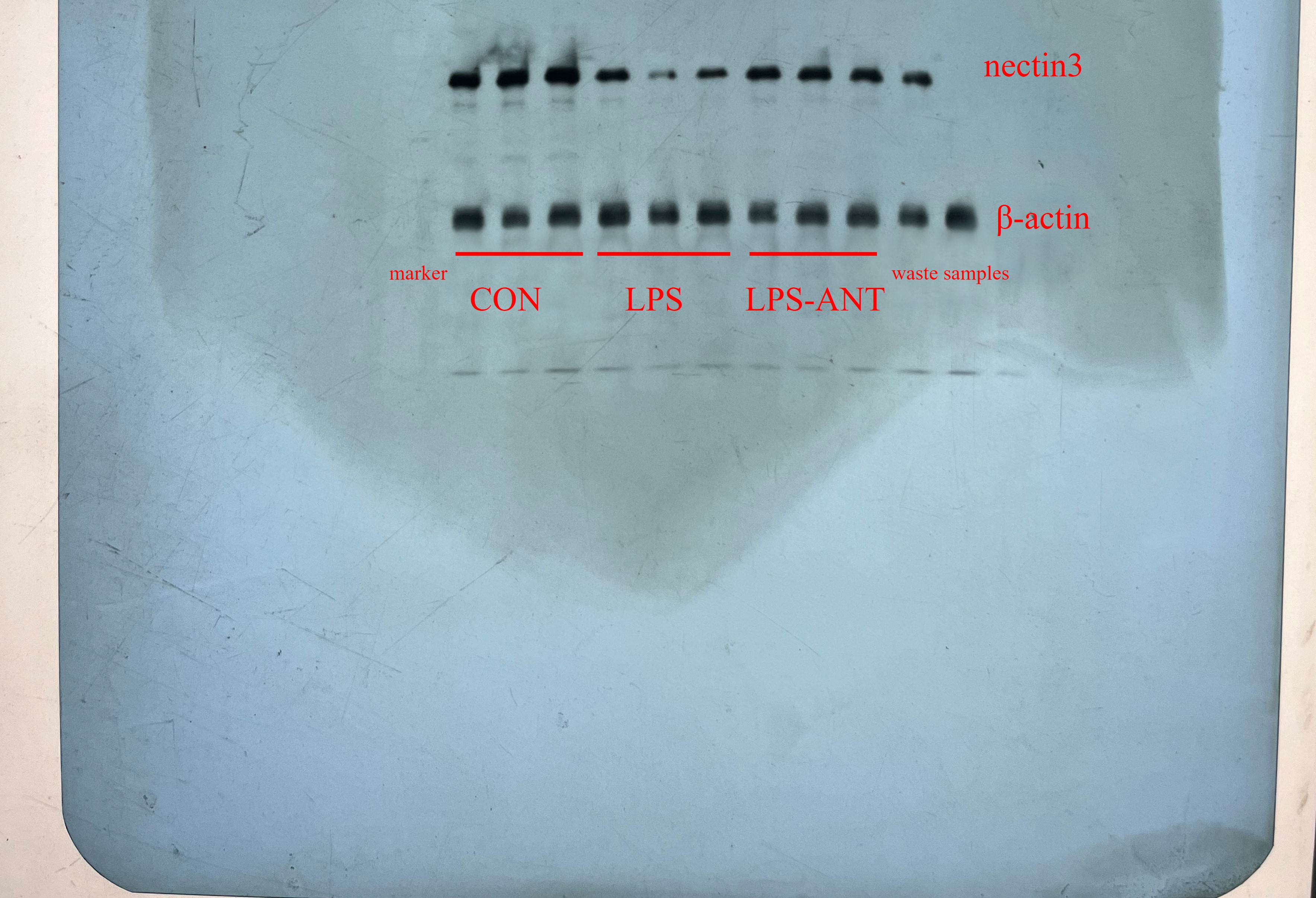

Supplement: Supplementary file 2 — Additional file 2. [file 12888_2023_4519_MOESM2_ESM.jpg]

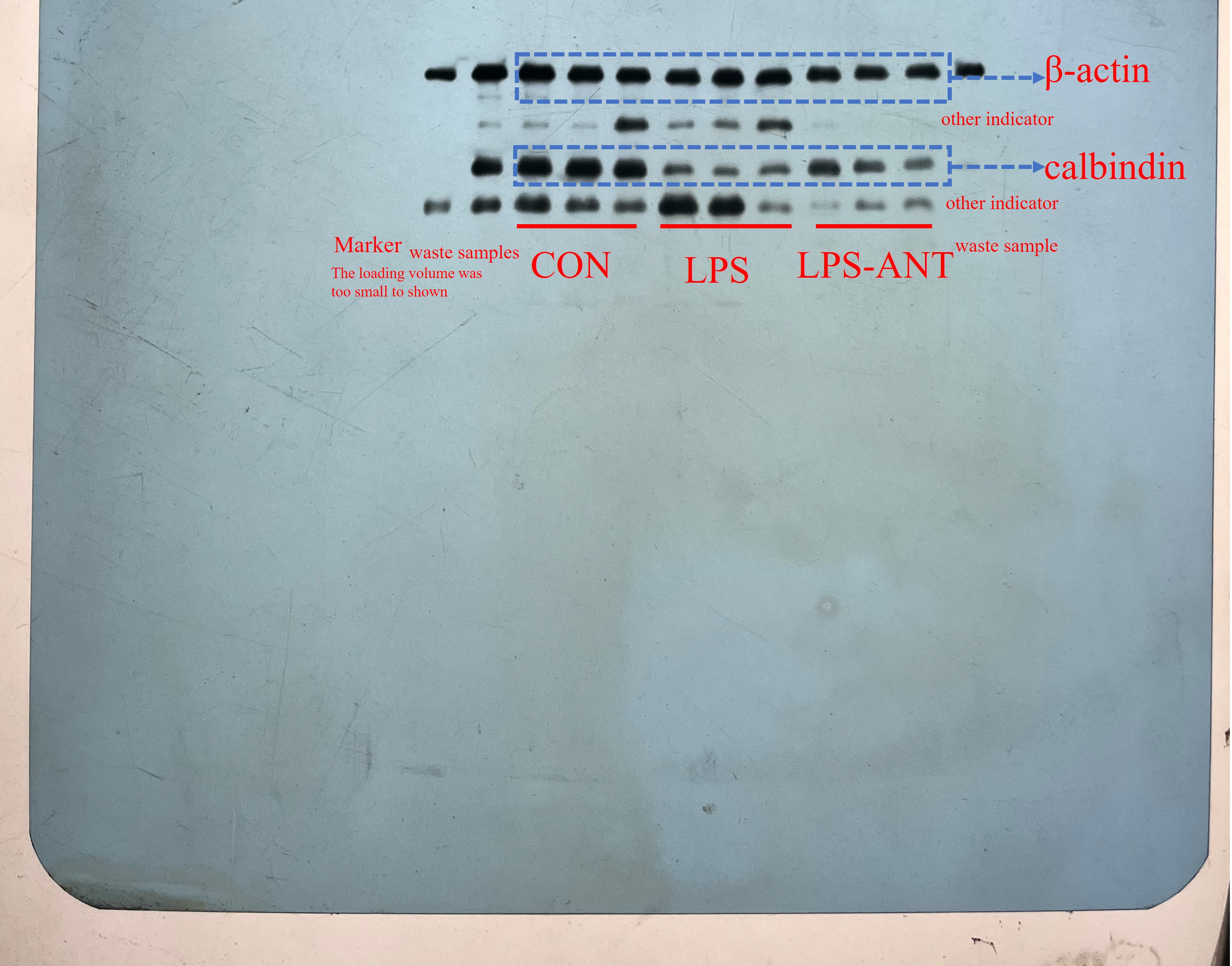

Supplement: Supplementary file 3 — Additional file 3. [file 12888_2023_4519_MOESM3_ESM.jpg]
